# Pre-Clinical Neuroprotective Evidences and Plausible Mechanisms of Sulforaphane in Alzheimer’s Disease

**DOI:** 10.3390/ijms22062929

**Published:** 2021-03-13

**Authors:** Jiyoung Kim

**Affiliations:** Center for Food and Bioconvergence, College of Agriculture and Life Sciences, Seoul National University, Seoul 08826, Korea; jiyoungkim1107@snu.ac.kr

**Keywords:** sulforaphane, Alzheimer’s disease, memory, amyloid-β, tau, inflammation, oxidative stress, neurodegeneration

## Abstract

Sulforaphane, a potent dietary bioactive agent obtainable from cruciferous vegetables, has been extensively studied for its effects in disease prevention and therapy. Sulforaphane potently induces transcription factor nuclear factor erythroid 2-related factor 2 (Nrf2)-mediated expression of detoxification, anti-oxidation, and immune system-modulating enzymes, and possibly acts as an anti-carcinogenic agent. Several clinical trials are in progress to study the effect of diverse types of cruciferous vegetables and sulforaphane on prostate cancer, breast cancer, lung cancer, atopic asthmatics, skin aging, dermatitis, obesity, etc. Recently, the protective effects of sulforaphane on brain health were also considerably studied, where the studies have further extended to several neurological diseases, including Alzheimer’s disease (AD), Parkinson’s disease, Huntington’s disease, amyotrophic lateral sclerosis, multiple sclerosis, autism spectrum disorder, and schizophrenia. Animal and cell studies that employ sulforaphane against memory impairment and AD-related pre-clinical biomarkers on amyloid-β, tau, inflammation, oxidative stress, and neurodegeneration are summarized, and plausible neuroprotective mechanisms of sulforaphane to help prevent AD are discussed. The increase in pre-clinical evidences consistently suggests that sulforaphane has a multi-faceted neuroprotective effect on AD pathophysiology. The anti-AD-like evidence of sulforaphane seen in cells and animals indicates the need to pursue sulforaphane research for relevant biomarkers in AD pre-symptomatic populations.

## 1. History of Sulforaphane Research in Brain Health

Sulforaphane (1-isothiocyanato-4-methylsulfonylbutane) is a potent dietary bioactive agent obtainable from cruciferous vegetables, such as broccoli, watercress, kale, cabbage, collard greens, brussels sprouts, bok choy, mustard greens, and cauliflower [1]. In cruciferous vegetables, sulforaphane is present as its precursor glucosinolate glucoraphanine [2]. When the tissues of cruciferous plants are processed by cutting, cooking, freezing, or mastication, glucoraphanine and an enzyme called myrosinase in the cruciferous vegetables are exposed to each other, which removes the glucose moiety on glucoraphanine and hydrolyzes glucoraphanine to sulforaphane [3]. β-Thioglucosidases occurring in the human gastrointestinal microbiome also convert precursor glucoraphanine into bioactive sulforaphane [4].

The study of the pharmacological effects of sulforaphane and cruciferous plants was started in 1992 by Zhang, Y. et al. [5]. They confirmed in rodent tissues that sulforaphane induces the expression of phase II detoxification enzymes, such as NAD(P)H:(quinone-acceptor) oxidoreductase 1 (NQO1) and glutathione S-transferases, indicating the possibility contributing to the detoxification mechanism of carcinogens and showing the possibility of chemoprevention [5]. Thereafter, the pharmacological potential of sulforaphane and cruciferous vegetables has been studied and published not only in cancer, but also in metabolic diseases, *Helicobacter pylori* infection, the nervous system, the cardiovascular system, liver, lungs, skin and even mortality. According to clinicaltrials.gov, clinical trials of 159 conditions to study the effects of sulforaphane on human health and diseases have been conducted or are in progress. In 1994, structural analogues of sulforaphane were synthesized, but none showed superior activity compared to sulforaphane [6], and no cases were applied to clinical studies.

The study on the neuroprotective effects of sulforaphane began in 2004 with studies showing the protective effects on neurons [7] and microglia [8] against oxidative stress via the activation of nuclear factor erythroid 2-related factor 2 (Nrf2), the transcription factor to induce the expression of detoxification, anti-oxidation, and immune system-modulating enzymes. Sulforaphane-induced hormetic activation of Nrf2 provides the possibility of reducing the wide range of human-related neurological pathologies in the experimental disease models on Alzheimer’s disease (AD) [9], Parkinson’s disease [10], Huntington’s disease [11], amyotrophic lateral sclerosis [12], multiple sclerosis [13], autism spectrum disorder (ASD) [14], and schizophrenia [15]. Now, sulforaphane studies are extended to various mechanisms via or not via Nrf2 and suggest the possibility of preventing or treating neurological diseases. Small-scale human clinical trials were conducted in patients with ASD and schizophrenia, and sulforaphane treatment reversed cognitive and behavior abnormalities that have been associated with ASD and schizophrenia [16,17,18,19,20]. In addition, the safety and efficacy of sulforaphane as an adjuvant to risperidone, an atypical antipsychotic agent, has recently been studied and no severe adverse events but improved irritability and hyperactivity symptoms in children with ASD were observed [21]. These findings suggest the possibility that sulforaphane reverses cognitive and behavior abnormalities in AD without severe adverse events. According to clinicaltrials.gov, there is a clinical trial underway in China using sulforaphane in patients with prodromal to mild AD (NCT04213391).

## 2. Evidence of Anti-AD Activity of Sulforaphane in Animals and Cells

Alzheimer’s disease is a slowly progressive neurodegenerative disease that currently has no effective treatment. The most discernible pathology that can identify AD is the extracellular formation of plaques produced by the accumulation of amyloid-β (Aβ) protein [22], and the formation of intraneuronal neurofibrillary tangles made of hyperphosphorylated and truncated tau proteins in cortical neurons [23]. These pathologies are known to occur decades before symptoms of AD appear [24]. Accumulation of Aβ and hyperphosphorylated and truncated tau proteins causes oxidative and inflammatory damage to brain tissue, which harms the function of neuronal synapses and induces neuronal degeneration, ultimately leading to symptoms of memory loss seen in AD [24]. 

Since there is no suitable treatment for AD, the overall goal of AD management is to reduce the incidence of disease in the target population and to ensure pre-symptomatic disease does not proceed to a later stage [25]. Several biomarkers are being studied to diagnose the likelihood of developing a disease early in the disease process, where prevention or treatment will be most effective, and to monitor a patient’s response to prevention and treatment [26]. Based on the biomarkers currently being studied for clinical practice [27,28,29,30], pre-clinical biomarkers (1) Aβ, (2) tau, (3) inflammation, (4) oxidative stress, (5) neurodegeneration, as well as (6) cognitive impairment were selected to investigate the pre-clinical anti-AD evidence of sulforaphane, and its effectiveness and plausible mechanisms. The characteristics of the AD-like cells and animal models cited in this review are described in detail in Table 1. Studies on transgenic mice [31,32,33,34,35] or cells carrying the gene mutations associated with AD [9,34], and animal models [36,37] or cells treated with Aβ [32,35,38,39,40,41,42,43] were reviewed (Table 2, Table 3, Table 4, Table 5 and Table 6). 

**Table 1:** Alzheimer’s disease (AD)-like animal and cell models cited in this review to examine the AD neuroprotective potential of sulforaphane.

Model	Description	Ref.
**transgenic animal models carrying gene mutations associated with AD**		
5×FAD mice	5×FAD mice were made to harbor five-transgenes APP_Swe_, APP_Florida_, APP_London_, PS1_M146L_, and PS1_L286V_. The resulting 5×FAD shows impaired memory and quickly constitute a major feature of AD amyloid pathology. 5×FAD has been proposed as a useful model for neurodegeneration and amyloid plaque formation induced by intraneuronal Aβ_42_.	[31,44]
3×Tg-AD mice	3×Tg-AD mice were designed to accommodate triple-transgenes APP_Swe_, PS1_M146V_, and tau_P301L_. 3×Tg-AD is the first model of the developed AD-like animal models to exhibit both plaque and tangle pathology. Consequently, 3×Tg-AD mice show synaptic damage and memory impairment.	[31,33,34,45]
APP/PS1 mice	APP/PS1 mice harbor double-transgenes APP_Swe_ and PS1_dE_. APP_Swe_/PS1_dE_ gene mutations are the Swedish-mutated APP gene combined with the exon-9-deleted PS1 gene. APP/PS1 mouse model exhibits amyloid plaque and memory impairment, recapitulating the onset and progression of early-onset familial AD.	[35,46]
PS1_V97L_ mice	PS1_V97L_ is a single-mutant transgenic mouse model harboring PS1_V97L_. It was generated by the report of a single missense mutation Val97Leu (V97L) of PS1 in a Chinese pedigree suffering from early-onset AD. Human Val97Leu mutant PS1 increases Aβ oligomers and tau phosphorylation level as well as AD-associated neuroinflammation and oxidative stress and finally causes spatial memory deficit in mice.	[32,47]
**cell models carrying gene mutations associated with AD**		
primary cortical neurons derived from 3×Tg-AD mice	Mouse primary cortical cells derived from 3×Tg-AD mice stably express APP_Swe_, PS1_M146V_, and tau_P301L_ and produce AD-associated high amount of Aβ, tau and p-tau.	[34]
mouse neuroblastoma N2a cells expressing APP_swe_	Mouse neuroblastoma Neuro2a cells stably expressing the human APP_Swe_ produce AD-associated high amount of Aβ and exhibit neuroinflammation and oxidative stress.	[9]
**AD-like animal models induced by Aβ**		
AD-like rat induced by Aβ_42_	SD rat model administered Aβ_42_ by i.c.v. injection shows AD-associated neuroinflammation and oxidative stress in its brain. This model also exhibits impaired spatial learning.	[37]
AD-like mice induced by Aβ_1–40_	This mouse model implemented the i.c.v. injection of Aβ_1–40_ shows impaired cognitive function.	[36]
**AD-like cell models induced by Aβ**		
murine cortical neurons treated with Aβ_42_	Rat primary cortical cells isolated from 18-day-old SD rat embryos exposed to Aβ_42_ show AD-associated tau hyperphosphorylation, damaged dendritic integrity and neuronal cell death.	[32]
human neuroblastoma SH-SY5Y cells treated with Aβ_25–35_	Human neuroblastoma SH-SY5Y cells exposed to Aβ_25–35_ show AD-associated oxidative stress and neuronal cell death.	[40,43]
murine neuroblastoma N2a cells treated with Aβ_1–42_	Mouse neuroblastoma Neuro2a cells exposed to Aβ_1–42_ show AD-associated neuronal cell death.	[41]
murine neuroblastoma N1E-115 cells treated with Aβ_1–42_	Mouse neuroblastoma N1E-115 cells exposed to Aβ_1–42_ show AD-associated neuronal cell death.	[41]
human microglia-like THP-1 cells treated with Aβ_1–42_	Differentiated human monocytic THP-1 cells, most closely resembling microglia, exposed to Aβ_1–42_ mimic AD-associated inflammatory microglial activation.	[38,39]
murine EOC-20 microglial cells treated with Aβ_1–42_	Mouse EOC-20 microglial cells exposed to Aβ_1–42_ mimic AD-associated damaged microglial phagocytosis.	[42]

i.c.v., intracerebroventricular; i.p., intraperitoneal; s.c., subcutaneously.

### 2.1. Sulforaphane and Aβ

Sulforaphane has been found to prevent Aβ production in the brains of AD-like animal models (Table 2). In the brains of PS1_V97L_ mice, single-mutant transgenic model harboring PS1_V97L_, as described in Table 1, sulforaphane treatment decreased the expression of β-secretase (BACE1) and presenilin-1 (PS-1), which are involved in the sequential proteolytic cleavage of amyloid-β protein precursor (AβPP) to Aβ peptides [32]. In the study with these mice, sulforaphane inhibited the generation of all types (monomer, trimer, tetramer, hexamer, nonamer, and dodecamer) of Aβ oligomers [32], which may be associated with the inhibition of BACE1 and PS-1 expression. In another study, depletion of Nrf2 increases BACE1 and a BACE1 mRNA-stabilizing antisense RNA (BACE1-AS) expression and Aβ production and worsens cognitive deficits, and suggesting that sulforaphane-mediated Nrf2 activation decreases BACE1 and BACE1-AS expression and Aβ production and ameliorates cognitive deficits in 5×FAD and 3×Tg-AD mice [31]. On the other hand, kinetics and computational studies on direct sulforaphane effect on BACE1 showed that sulforaphane has selective and non-competitive BACE1 inhibitory activity [48].

**Table 2:** The effects of sulforaphane on Aβ and tau in Alzheimer’s disease (AD)-like models.

Model	Sulforaphane Dose	Findings	Ref.
5×FAD mice	every other day 10 mg/kg i.p. for 2 months	in cortex: (1) reduced the numbers of Aβ plaques/mm^2^ in cerebral cortex: (1) reduced Aβ_1–40_ and Aβ_1–42_ levels (2) reduced BACE1 protein expression (3) reduced BACE1 and BACE1-AS transcript (4) increased NQO1 transcript and protein expression (maybe through Nrf2 activation) in hippocampus: (1) reduced the numbers of Aβ plaques/mm^2^	[31]
3×Tg-AD mice	every other day 5 or 10 mg/kg i.p. for 2 months	in cortex: (1) reduced Aβ40 and Aβ42 in cerebral cortex: (1) reduced p-tau level (2) reduced BACE1 mRNA and protein expression (3) increased HO-1 mRNA and protein expression (maybe through Nrf2 activation) in hippocampus: (1) reduced p-tau pathology	[31]
	daily 10 or 50 mg/kg p.o. 6 days/week for 2 months	in whole cortex and the fifth layer of the cortex: (1) decreased AβPP/Aβ level (2) decreased tau level (3) increased CHIP level in hippocampus: (1) decreased AβPP, polymeric Aβ, and monomeric Aβ (2) did not alter AβPP mRNA level (3) decreased tau and p-tau (4) did not alter tau mRNA level (5) increased CHIP and HSP70	[34]
APP/PS1 mice	daily 25 mg/kg p.o. for 5 months	in cerebral cortex: (1) protected against the increment of Aβ plaques (2) up-regulated p75 NTR (3) increased levels of Ace-H3K9 and Ace-H4K12 (4) reduced expression of HDAC1 and 3 suggested to contribute to up-regulation of p75 NTR	[35]
PS1_V97L_ mice	daily 5 mg/kg i.p. for 4 months	in brain: (1) inhibited the generation of all types of Aβ oligomers (monomer, trimer, tetramer, hexamer, nonamer, and dodecamer) (2) alleviated tau hyperphosphorylation (3) decreased the expression levels of BACE1 and PS1	[32]
primary cortical neurons derived from 3×Tg-AD mice	10 μM for 6 h	(1) decreased Aβ (2) decreased tau (3) increased CHIP in the absence of CHIP expression: (1) failed to decrease Aβ (2) failed to decrease tau	[34]
mouse neuroblastoma N2a cells expressing APP_swe_	1.25 or 2.5 μM for 48 h	in both cells and culture medium: (1) decreased levels of Aβ_1–40_ and Aβ_1–42_	[9]
murine cortical neurons treated with Aβ_42_	0.01, 0.03 or 0.1 μM pre-treatment for 30 min followed by Aβ_42_	(1) decreased hyperphosphorylation of tau	[32]
sulforaphane and Aβ		analyzed by mass spectrometry: (1) showed a 1:1 complex of [Aβ+sulforaphane] (2) formed three different [Aβ+sulforaphane] complexes due to covalent binding of sulforaphane to Aβ at three different sites (3) sulforaphane bound to free NH_2_ groups (N-terminal amino acid and lysines) in Aβ	[49]
sulforaphane and BACE1		analyzed by fluorescence resonance energy transfer: (1) sulforaphane shown selective and non-competitive BACE1 inhibitory activity	[48]

i.p., intraperitoneally; p.o., *per os* (by mouth); Ace-H3K9, acetylated histone 3 lysine 9; Ace-H4K12, acetylated histone 4 lysine 12.

In the brains of APP/PS1 mice, sulforaphane decreased the increment of Aβ plaques and the mechanism underlying these effects was suggested to be associated with increased expression of p75 neurotrophin receptor (p75 NTR) via reducing the expression of histone deacetylase (HDAC) 1 and 3 [35]. p75 NTR has been reported to be significantly reduced in the brains of AD patients [50,51] and p75 NTR-ectodomain has been reported to reduce local Aβ plaques in APP/PS1 mice [52].

In the hippocampus of 3×Tg-AD mice, oral gavage of sulforaphane reduced the monomeric and polymeric forms of Aβ [34]. In this animal model, it was found that sulforaphane treatment increased the levels of heat shock protein 70 (HSP70) and the C-terminus of HSP70-interacting protein (CHIP), previously reported to influence Aβ metabolism [53], and the sulforaphane-mediated reduction in Aβ was dependent on CHIP expression, suggesting that sulforaphane clears the monomeric and polymeric forms of Aβ by upregulating CHIP [34]. Even in primary cortical neurons derived from 3×Tg-AD mice, sulforaphane increased CHIP and decreased Aβ [34]. In addition to that, sulforaphane treatment in mouse neuroblastoma N2a cells expressing APPswe decreased the levels of Aβs in both the cells and the culture medium [9]. 

Aβ aggregation studies with sulforaphane showed that Aβ is less prone to aggregate when sulforaphane is present [49]. Mass spectrometry experiments suggested a possible mechanism for sulforaphane’s effects, by showing that sulforaphane binds to free NH_2_ groups (N-terminal amino acid and lysines) in Aβ and forms three different 1:1 complexes of Aβ+sulforaphane [49]. 

Together, the above observations suggest that sulforaphane not only inhibits Aβ production by decreasing the expression of BACE1 and PS1 [32] but also clears Aβ molecules by inducing the expression of the HSP co-chaperone CHIP [34]. Sulforaphane-mediated activation of the Nrf2/ARE pathway negatively regulates BACE1 expression [31]. Sulforaphane-mediated upregulation of the p75 NTR at least in part through decreased expression of HDAC 1 and 3 may also contribute to reduce the increase in Aβ plaque [35]. Although further investigations are required, sulforaphane has the potential to inhibit Aβ aggregation by binding directly to Aβ in the brains of AD patients [49].

### 2.2. Sulforaphane and Tau

Tau is the main component of neurofibrillary tangles in AD and is a causative factor for AD [54,55]. Sulforaphane showed the ability of reducing protein levels of tau [34] and p-tau [31,32,34] in 3×Tg-AD and PS1_V97L_ mice. In the study using 3×Tg-AD mice, sulforaphane treatment reduced tau and p-tau at the protein level, but not mRNA, suggesting post-translational modifications in tau expression [34]. Sulforaphane treatment has been observed to increase the level of HSP70 and CHIP, which has a unique binding affinity for tau and is known to play a role in tau ubiquitination and proteasome targeting for tau degradation [34,56]. In murine cortical neurons treated with Aβ oligomers, sulforaphane also decreased the Aβ-induced hyperphosphorylation of tau [32]. 

Although further studies are required to understand how sulforaphane affects tau biology, Aβ, Nrf2 lack, oxidative stress, and inflammation are known factors to promote tau phosphorylation [57,58,59,60], thus sulforaphane’s effect on inhibiting the production of Aβ [9,31,32,34,35,61], activating Nrf2 [9,10,31,38], and alleviating oxidative stress [9,32,37,40,43] and inflammation [9,32,37,38,39,42] may inhibit tau phosphorylation. The effects of sulforaphane on tau were overviewed in Table 2.

### 2.3. Sulforaphane and AD-Associated Inflammatory Biomarkers 

Sulforaphane also affects inflammatory biomarkers associated with AD, as below in AD-like cell and animal models (Table 3). For example, sulforaphane treatment of PS1_V97L_ mice resulted in decreased levels of interleukin (IL)-1β in brain tissue and tumor necrosis factor alpha (TNF-α) in the plasma [32]. Sulforaphane treatment of AD-like rat induced by Aβ reduced neuroinflammation measured through the reduction of TNF-α and IL-1β [37]. In mouse neuroblastoma N2a cells expressing APPswe, sulforaphane decreased the levels of IL-1β, IL-6, cyclooxygenase-2 (COX-2), inducible nitric oxide synthase (iNOS), and nuclear factor (NF)-κB p-p65 [9]. In human microglia-like THP-1 cells treated with Aβ, sulforaphane suppressed the activation of NLRP3 (NLRP3: NOD-like receptor, leucine-rich repeat (LRR), and pyrin domain-containing 3) inflammasome [38]. Aβ activation of the NLRP3 inflammasome in microglia has been reported to contribute to IL-1β maturation and subsequent inflammatory responses [62]. Mice that are deficient in NLRP3 are mostly protected from amyloid pathology, suppression of synaptic plasticity, and abnormal cognitive functions [62,63]. Thus, sulforaphane might play a role in reducing Aβ-induced neuroinflammation, amyloid pathology, and synaptic and cognitive dysfunction via inhibition of NLRP3. In human microglia-like THP-1 cells treated with Aβ, sulforaphane also suppressed subsequent IL-1β secretion via the dephosphorylation of signal transducers and activators of transcription-1 (STAT-1), and the activation of the Nrf2-regulated heme oxygenase-1 (HO-1) pathway [38]. microRNA (miRNA)-146a is a potential AD biomarker that appears to be selectively upregulated in the temporal cortex and hippocampus in AD [64,65] and sulforaphane treatment in THP-1 cells decreased the Aβ-induced expression of miRNA-146a [38]. 

**Table 3:** The effects of sulforaphane on Alzheimer’s disease (AD)-associated inflammatory biomarkers in AD-like models.

Model	Sulforaphane Dose	Findings	Ref.
PS1_V97L_ mice	daily 5 mg/kg i.p. for 4 months	in brain: (1) decreased IL-1β and TNF-α	[32]
AD-like rat induced by Aβ_42_	daily 5 mg/kg i.p. for 7 days	in brain: (1) decreased IL-1β and TNF-α	[37]
mouse neuroblastoma N2a cells expressing APP_swe_	1.25 or 2.5 μM for 48 h	(1) decreased IL-1β and IL-6 (2) decreased COX-2 and iNOS (3) reduced NF-κB p-p65	[9]
human microglia-like THP-1 cells treated with Aβ_1–42_	5 μM pre-treatment for 30 min followed by Aβ_1–42_	(1) inhibited IL-1β secretion (2) inhibited miRNA-146a production (3) reduced NLRP3 inflammasome (4) reduced STAT-1 activation (5) induced HO-1 gene expression 6) increased nuclear Nrf2 levels	[38]
	5 μM pre-treatment for 30 min followed by Aβ_1–42_	(1) decreased IL-1β and TNF-α (2) attenuated MerTK reduction (3) inhibited NF-κB signaling (4) decreased intracellular Ca^2+^ levels	[39]
murine EOC-20 microglial cells treated with Aβ_1–42_	5 μM co-treatment with Aβ_1–42_ for 24 h	(1) induced the phagocytic activity (2) induced FPR2 expression	[42]

i.p., intraperitoneally.

Meanwhile, it has been published that sulforaphane stimulates phagocytosis of immune cells [66,67]. According to recent research results, sulforaphane treatment was able to significantly induce the expression of formyl peptide receptor (Fpr2), which is known to regulate microglial phagocytosis and recovers the phagocytic activity of murine EOC-20 microglial cells decreased by Aβ oligomers [42]. In THP-1 cells, sulforaphane treatment attenuated the effects of Aβ induction, by reducing excessive intracellular Ca^2+^ levels and blocking NF-κB to rescue the decrease in Mer tyrosine kinase (MerTK) activity that is necessary for amyloid-stimulated phagocytosis [39]. Thus, sulforaphane inhibited NF-κB-mediated IL-1β and TNF-α secretion in THP-1 cells treated with Aβ [39]. As activation of Nrf2, an important sulforaphane target protein, suppresses the hyperactivation of microglia and protects against inflammatory disorders [68,69], sulforaphane-mediated activation of Nrf2 might also contribute to reducing the levels of AD-associated inflammatory biomarkers.

### 2.4. Sulforaphane and AD-Associated Oxidative Stress Biomarkers

Oxidative damage to brain tissue caused by accumulation of Aβ and tau proteins impairs the function of neuronal synapses, induces neuronal degeneration, and eventually causes the symptoms of memory loss seen in AD [24]. Sulforaphane inhibits the levels of AD-associated oxidative stress biomarkers in AD-like models (Table 4). Sulforaphane augmented the cell’s capacity for antioxidant defense by activating Nrf2 in N2a cells expressing APPswe [9] and in SH-SY5Y cells treated with Aβ [40,43]. In N2a cells expressing APPswe, this occurs by promotion of Nrf2 nuclear translocation by decreasing DNA methylation levels of the Nrf2 promoter [9]. In these cells, sulforaphane also caused decreased levels of reactive oxygen species (ROS) and malondialdehyde (MDA) and increased superoxide dismutase (SOD) activity [9]. Even in PS1_V97L_ mice and AD-like rat induced by Aβ, sulforaphane increased GSH levels and decreased MDA levels [32,37]. Crude juices of broccoli sprouts containing sulforaphane in SH-SY5Y cells treated with Aβ reduced oxidative stress by intracellular increase of glutathione (GSH), HO-1, thioredoxin (Trx), and thioredoxin reductase (TrxR) as well as NQO-1 activity, likely through the Nrf2 signaling pathway [43]. Together, these studies indicate that sulforaphane treatment attenuates biomarkers of oxidative stress that are associated with AD.

**Table 4:** The effects of sulforaphane on Alzheimer’s disease (AD)-associated oxidative stress biomarkers in AD-like models.

Model	Sulforaphane Dose	Findings	Ref.
PS1_V97L_ mice	daily 5 mg/kg i.p. for 4 months	in brain: (1) increased GSH (2) decreased MDA	[32]
AD-like rat induced by A*β*_42_	daily 5 mg/kg i.p. for 7 days	in brain: (1) increased GSH (2) decreased MDA	[37]
mouse neuroblastoma N2a cells expressing APP_swe_	1.25 or 2.5 μM for 48 h	(1) decreased ROS and MDA (2) increased SOD activity (3) upregulated Nrf2 expression and promoted Nrf2 nuclear translocation via decreasing DNA methylation levels of the Nrf2 promoter	[9]
human neuroblastoma SH-SY5Y cells treated with A*β*_25–35_	1–5 μM pre-treatment for 30 min followed by A*β*_25–35_	(1) inhibited ROS production and subsequent oxidative damages (2) increased NQO1, HO-1 and g-GCS (3) activated Nrf2	[40]
	1 μM co-treatment with A*β*_25–35_	(1) increased GSH (2) increased Trx expression (3) increased HO-1 and TrxR expression 4) increased NQO1 activity (5) activated Nrf2	[43]

i.p., intraperitoneally; p.o., *per os* (by mouth); γ-GCS, γ -glutamylcysteine synthetase.

### 2.5. Sulforaphane and AD-Associated Biomarkers of Synaptic Damage and Neurodegeneration

Sulforaphane inhibits the levels of AD-associated biomarkers of synaptic damage and neurodegeneration in AD-like models, as well as inhibiting Aβ-induced neuronal cell death (Table 5). For example, the brain of 3×Tg-AD has low levels of brain-derived neurotrophic factor (BDNF), a neurotrophin that supports the survival of existing neurons and encourages the growth and differentiation of new neurons and synapses, but sulforaphane has been found to restore normal levels of BDNF of 3×Tg-AD mice [33]. In the frontal cortex of 3×Tg-AD mice, sulforaphane increased levels of neuronal and synaptic biomarkers, including: microtubule-associated protein 2 (MAP2), a dendritic marker and structural protein present in neurons; synaptophysin, a pre-synaptic protein; and postsynaptic density protein-95 (PSD-95), a membrane-associated protein located in neural postsynaptic densities [33]. It was reported that sulforaphane inhibits HDAC activity and increases histone-tail acetylation in 3×Tg-AD mice, thereby increasing BDNF levels and enhancing activation of the BDNF-TrkB signaling pathways, which might further facilitate neuronal differentiation and growth, promote survival of neurons, and induce synaptic plasticity and long-term potentiation [33]. Thus, sulforaphane might improve neuronal and cognitive functions, at least in part, by increasing BDNF levels epigenetically in BDNF-deficient neuronal disorders such as AD [33]. 

**Table 5:** The effects of sulforaphane on Alzheimer’s disease (AD)-associated biomarkers of synaptic damage and neurodegeneration such as cell death in AD-like models.

Model	Sulforaphane Dose	Findings	Ref.
3×Tg-AD mice	10 or 50 mg/kg p.o., 6 days/week for 2 months	in the frontal cortex: (1) increased MAP2, synaptophysin, and PSD-95 (2) activated TrkB signaling pathway in the cortex and hippocampal CA1: (1) increased BDNF levels	[33]
murine cortical neurons treated with A*β*_42_	0.01, 0.03 or 0.1 μM pre-treatment for 30 min followed by A*β*_42_	(1) protected against cell death (2) rescued dendritic integrity	[32]
human neuroblastoma SH-SY5Y cells treated with A*β*_25–35_	2 μM pre-treatment for 3 h followed by A*β*_25–35_	(1) protected against cell death (2) up-regulated p75 NTR (3) increased levels of Ace-H3K9 and Ace-H4K12 (4) reduced expression of HDAC1 and 3 suggested to contribute to up-regulation of p75 NTR	[35]
	1–5 μM pre-treatment for 30 min followed by A*β*_25–35_	(1) protected against cell death (2) reduced Bax/Bcl-2 (3) reduced activation of JNK	[40]
	1 μM co-treatment with A*β*_25–35_	(1) protected against cell death (2) increased HSP70	[43]
murine neuroblastoma N2A cells treated with A*β*_1–42_	2.5 μM pre-treatment for 18 h followed by A*β*_1–42_	(1) protected against cell death (2) sulforaphane effect dependent on proteasome activity	[41]
murine neuroblastoma N1E-115 treated with A*β*_1–42_	5 μM pre-treatment for 18 h followed by A*β*_1–42_	(1) protected against cell death	

p.o., per os (by mouth).

Several studies have reported that sulforaphane prevented Aβ-induced cell death in neuron-like cells (Table 5) [32,35,40,41,43]. Treatment with broccoli sprouts juices containing sulforaphane overexpressed HSP70 and exerted a protective action against the cytotoxicity and cell death Aβ induced [43]. Sulforaphane rescued the dendritic length of the cortical neurons, which was reduced following Aβ incubation [32]. These studies indicate that sulforaphane treatment might attenuate AD pathology, as evidenced by its effect on pre-clinical biomarkers associated with synaptic damage and neurodegeneration.

### 2.6. Sulforaphane and Cognitive Impairment in AD-Like Animal Models

Sulforaphane ameliorated cognitive impairment in several transgenic AD-like animal models, including 5×FAD mice [31], 3×Tg-AD mice [31,34], APP/PS1 mice [35], and PS1_V97L_ mice [32]. Additionally, sulforaphane treatment improved cognitive function in AD-like mouse and rat model induced by the administration of Aβ [36,37]. Although it is difficult to explain the exact mechanism, it is highly likely that the pharmacological effects of sulforaphane on the previously described pre-clinical AD biomarkers, Aβ, tau, inflammation, oxidative stress, and neurodegeneration have led to improvements in cognitive impairment. The types of AD-like models, the dose of sulforaphane, and cognitive recovery findings for sulforaphane are summarized in Table 6. These observations suggest that sulforaphane might be able to ameliorate cognitive impairment in AD patients.

**Table 6:** The effects of sulforaphane on cognitive impairment in Alzheimer’s disease (AD)-like animal models.

Model	Sulforaphane Dose	Findings	Ref.
5×FAD mice	every other day 10 mg/kg i.p. for 2 months	ameliorated cognitive deficits (Morris water maze tests and passive avoidance tests)	[31]
3×Tg-AD mice	every other day 5 or 10 mg/kg i.p. for 2 months	ameliorated cognitive deficits (Morris water maze tests)	[31]
	daily 10 or 50 mg/kg p.o. 6 days/week for 2 months	ameliorated memory deficit (novel object/location recognition tests and contextual fear conditioning tests)	[34]
APP/PS1 mice	daily 25 mg/kg p.o. for 5 months	ameliorated cognitive dysfunction (open field and Morris water maze tests)	[35]
PS1_V97L_ mice	daily 5 mg/kg i.p. for 4 months	alleviated cognitive deficit (Morris water maze tests)	[32]
AD-like mice induced by A*β*_1–40_	daily 30 mg/kg i.p. for 6 days	ameliorated cognitive function (Y-maze and passive avoidance behavior tests)	[36]
AD-like rat induced by A*β*_42_	daily 5 mg/kg i.p. for 7 days	improved spatial learning	[37]

i.p., intraperitoneally; p.o., *per os* (by mouth).

## 3. Conclusions and Future Perspectives

The anti-AD-like activity of sulforaphan has been identified in six different animal models and eight different cell models (Table 1, Table 2, Table 3, Table 4, Table 5 and Table 6). In animal models, four animal models produced by modifying AD-related genes in an animal model to create the pathology of human AD, and a mouse and a rat model made by direct intracerebroventricular (i.c.v.) injections of Aβ into the brain, which is known to induce AD-like pathology in the brain, were used to study the anti-AD-like activity of sulforaphan. In animal models, sulforaphane was administered daily or every other day, for oral administration (per os, p.o.) for 2 to 5 months, and for intraperitoneal injection (i.p.) for 7 days to 4 months. In the case of oral administration, a dose of 10–50 mg/kg, in the case of i.p. injection, 5–10 mg/kg was tried to show the anti-AD-like activity of sulforaphane. In cell models, sulforaphane showed anti-AD-like effectiveness under the conditions of 0.01 μM–10 μM, 0.5–48 h treatment.

**Figure 1:**
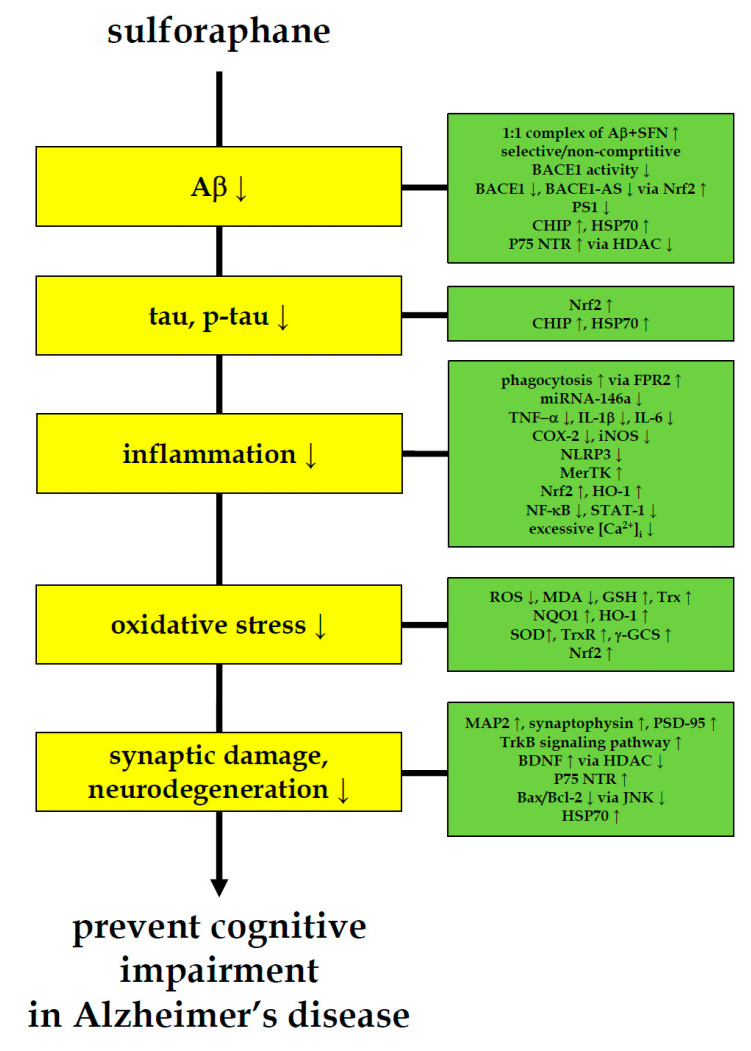
Multi-faceted anti-AD-like activity of sulforaphane. Sulforaphane ameliorates cognitive impairment and decreases the level of AD biomarkers for amyloid-β, tau, inflammation, oxidative stress and neurodegeneration in AD-like animal and cell models. These anti-AD-like evidences of sulforaphane indicate the need to pursue sulforaphane research in pre-symptomatic AD patients.

It has been reported that AD already has pre-symptomatic pathologies in the brain 10–15 years before cognitive decline [26]. In order to apply food-derived physiologically active compounds such as sulforaphane to control AD, interventions that prevent or delay the initial onset of symptoms are necessary. For this, research to find and develop early diagnostic biomarkers capable of actively diagnosing patients with AD is essential. In 2018, the National Institute on Aging (NIA) and Alzheimer’s Association (AA) proposed a new framework to define AD diagnosis criteria based on pre-symptomatic biomarkers [70]. The research framework focuses on the diagnosis of AD with biomarkers grouped into those of Aβ deposition, pathologic tau, and neurodegeneration [70]. In this review, pre-clinical biomarkers (1) Aβ, (2) tau, (3) inflammation, (4) oxidative stress, and (5) neurodegeneration were selected based on the pre-symptomatic biomarkers currently being studied and suggested for clinical practices [27,28,29,30], and the effects and the mechanisms of sulforaphane on them were overviewed (Figure 1 and Table 2, Table 3, Table 4, Table 5 and Table 6). Most importantly, sulforaphane can prevent the production of Aβ and tau [9,31,32,34,35,48], which are the main causative factors for AD. Sulforaphane can inhibit AD-associated inflammation [9,32,37,38,39,42], oxidative stress [9,32,37,40,43], and neurodegeneration [32,33,35,40,41,43]. Thus far, multi-target effects of sulforaphane in many different cells and animal models suggest that sulforaphane consumption may reduce AD risk, raising the possibility of effective preventive strategies.

In one recent clinical trial, in which schizophrenic patients were administered sulforaphane-rich broccoli sprout extract (Kagome Co., Ltd., Nagoya, Japan), three tablets of sulforaphane per day (consisting of 30 mg of sulforaphane-glucosinolate per tablet) for 8 weeks resulted in improved cognitive function in schizophrenic patients [17]. In another clinical trial, administration of sulforaphane derived from broccoli sprouts (Johns Hopkins University, MD, USA) consisting of 50–150 μmol or 8.86–26.59 mg per day for 18 weeks improved behavior in young men with ASD [18]. Many sulforaphane supplements on the market are prepared in various ways from cruciferous plants, and the dosage, safety, and efficacy have not been verified. Among them, Avmavol^®^ (a sulforaphane-producing dietary supplement) was studied to investigate (1) the dose-response evaluation of sulforaphane, (2) the bioavailability and mucosal bioactivity in healthy subjects, (3) the treatment effects for youth with ASDs, (4) the effects on autistic people living in New Jersey, USA, and (5) the effects on schizophrenia [71].

In-depth human studies are needed on the medical benefits of a sulforaphane-rich broccoli sprout diet. The association between the Keap1 and Nrf2 genes and cognitive function in patients with schizophrenial was studied, with an epistatic interaction between Nrf2 and Keap1 gene variants on cognitive impairment (e.g., working memory and processing speed) [72]. Since sulforaphane modifies Keap1 structure and activates Nrf2 [68], these findings suggest that the Keap1-Nrf2 system, which regulates oxidative stress and inflammation, may play a role in the cognitive impairment observed in schizophrenia. In order to study the medical benefits of a sulforaphane-rich broccoli sprout diet on cognitive impairment in AD, further studies on the intake of sulforaphane-rich broccoli sprout diet or sulforaphane in subjects at high risk of AD are needed. Besides cognitive impairment, a sulforaphane-rich broccoli sprout diet reduced colonization and attenuates gastritis in people infected with *Helicobacter pylori* [73] and sulforaphane-rich broccoli sprout beverages modulated the detoxification metabolism of airborne-pollutants in the body, thereby reducing their associated health risks [74]. It has also been reported that daily intake of a sulforaphane-rich broccoli sprout for 4 weeks improved defecation bowel habits in human subjects [75]. 

In 2003, the World Health Organization (WHO) International Agency for Research on Cancer (IARC) published a chapter on cruciferous vegetables as recommendations for cancer prevention. Whether in the form of a sulforaphane supplement or in large amounts of cruciferous vegetables, WHO recommended not to consume large amounts. It is generally not advisable for individuals to consume a diet that focuses on only one food type and should be encouraged to consistently consume a variety of cruciferous vegetables as part of their diet. On the other hand, several clinical trials showed little to no side effects when reasonable amounts of cruciferous vegetables containing sulforaphane were consumed [1]. 

Evidences from several animal- and cell-based studies indicate the need to pursue sulforaphane research in pre-symptomatic AD patients, which is challenging. The discovery and development of practical and inexpensive biomarkers that can diagnose pre-symptomatic AD patients is essential. Genetic polymorphism, intestinal microbiome, and body mass index (BMI) may affect the bioavailability of sulforaphane from cruciferous vegetable consumption [76,77,78,79,80,81,82,83], and through detailed follow-up studies, predictable response groups of sulforaphane might be selected for randomized controlled trial. The increase in pre-clinical evidence consistently suggests that sulforaphane has a multi-faceted neuroprotective effect on AD pathophysiology and prevents the progression of the disease.

## Data Availability

Not applicable
